# A Rare Case Report of Facial Talon Cusp: 1.5‐Year Follow‐Up and Minireview of the Literature

**DOI:** 10.1155/crid/5577057

**Published:** 2026-05-31

**Authors:** Chuhong Ouyang, Jingwen Chen, Mingshu Huang

**Affiliations:** ^1^ Department of Endodontics, Stomatological Hospital of Southern Medical University, Guangzhou, Guangdong Province, China; ^2^ Department of Radiology, Stomatological Hospital of Southern Medical University, Guangzhou, Guangdong Province, China

**Keywords:** anterior teeth, facial talon cusp, management, permanent dentition, resin restoration

## Abstract

**Background:**

Talon cusp usually refers to the abnormal cusp‐like protrusion on labial/facial or lingual/palatal surface of anterior teeth, and the labial one is rarely seen. Labial talon cusps might be associated with caries susceptibility, occlusal interference, and esthetic problems. Here we showed the treatment process of a facial talon cusp and followed it for 1.5 years.

**Case Report:**

We report a case with a labial small triangular projection on the right maxillary permanent central incisor of an 8‐year‐old girl, which was diagnosed as Stage 1 talon cusp according to Mayes classification and consequently treated by grinding and flowable resin restoration, achieving satisfactory appearance and maintaining vitality of the pulp. One and a half years′ follow‐up showed the tooth developed well.

**Conclusions:**

The present paper demonstrates the clinical management of a facial talon cusp emphasizing minimally invasive and preventive principles. Furthermore, we summarize and propose the therapeutic strategy of labial talon cusp to offer increasing understanding according to a total of 20 cases reporting 26 labial talon cusps searched from the PubMed/Web of Science.

## 1. Introduction

Talon cusp is named due to its resemblance to eagle′s talon in shape from an occlusal view [1]. It is an accessory cusp‐like structure composed of normal enamel, dentin, and varying extension of pulp tissue on anterior teeth in both primary and permanent dentition [2, 3], which can occur on labial/facial and/or lingual/palatal surface. Yet there is not an explicit definition. In some literature, a ridge continuous with and protruding from the tooth surface was also recognized as talon cusp, which, however, is inconsistent with its name in form from the incisal view. Therefore, we suppose only the cusp standing away parallelly from the tooth surface, that is to say, with a developmental groove or fissure present where the cusp joins the tooth surface [4, 5] mimicking an appearance of eagle′s talon from the occlusal view, can be assumed as talon cusp.

Far more rare than lingual talon cusp [6, 7], labial talon cusp is extremely rare for little epidemiological information available. There were only dozens of labial talon cusps reported in the literature. It is essential to record every labial talon cusp case encountered in clinical practice to enlarge the database of this particular abnormality. In fact, facial and lingual talon cusps might not be the same trait because of their possible differences in etiology and pattern of occurrence [8, 9], yet they were discussed as a singular entity with regard to form. So this paper only targets facial talon cusp without involving lingual talon cusp since their illegible relevance. In 2007, Mayes categorized labial talon cusp into three types according to their occlusogingival length [6] (Table 1).

**Table 1:** Mayes classification of labial talon cusp.

Type	Description
Stage 1	The slightest form, consists of a slightly raised triangle on the labial surface of an incisor extending the length of the crown, but not reaching the cementoenamel junction or the incisal edge. Adjusting the tooth, or the light source, to cause shadows to form may be necessary to observe the cusp, which can be palpated.
Stage 2	The moderate form, is a raised triangle on the labial surface of an incisor that extends the length of the crown, does not reach the cementoenamel junction, but does reach the incisal edge. Labial talon cusps in this stage can be observed clearly and palpated easily.
Stage 3	The most extreme form, is a free‐form cusp extending from the cementoenamel junction to the incisal edge on the labial surface of an incisor.

Herein, by using Mayes classification system, a case diagnosed as Stage I labial talon cusp on the maxillary right central incisor is reported to record such a rare occurrence. Additionally, occurrence pattern, effect on eruption, and managements of labial talon cusp are also discussed, along with a summary of similar previously reported cases.

## 2. Case Report

**Figure 1:**
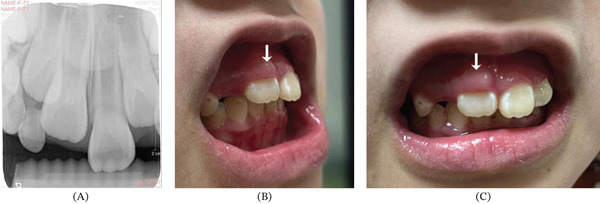
X‐ray image before eruption and clinical photographs under eruption of the Tooth #11. (A) The germ of the Tooth #11 below the alveolar crypt. (B) Lateral view of gum swelling during eruption of the Tooth #11. (C) Anterior view of gum swelling during eruption of the Tooth #11. White arrows indicate firm node‐like gingiva.

**Figure 2:**
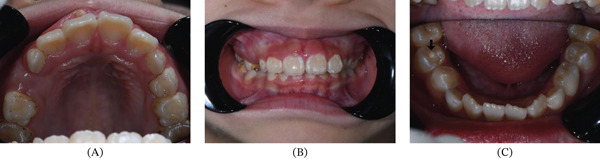
Clinical photographs after eruption of the Tooth #11. (A) Occlusal view of upper dentition. (B) Anterior view of dentition. (C) Occlusal view of lower dentition. Black arrow indicates an abnormal central cusp on occlusal surface of Tooth #45.

An 7‐year‐old girl was referred to the Department of Endodontics, Stomatological Hospital of Southern Medical University with the chief complaint of delayed eruption of upper right anterior teeth. The upper primary right incisor had fallen out for more than 1 year. Radiographic examination showed the right incisor has developed root to 2/3 its final length and was beneath the alveolar crypt, which indicated the teeth were in Nolla Stage 8 ready for eruption (Figure 1a). The patient was suggested clinical observation and periodic follow‐up. Four months later, the patient came with the erupted right incisor and asymptomatic anterior gum swelling (Figure 1b,c). Given the teeth under eruption, the suggestion was continuous observation. After another half a year, the girl occurred with an extra labial cusp adjacent to the gingival edge on the right incisor and complained about gum bleeding when brushing the teeth (Figure 2a,b).

**Figure 3:**
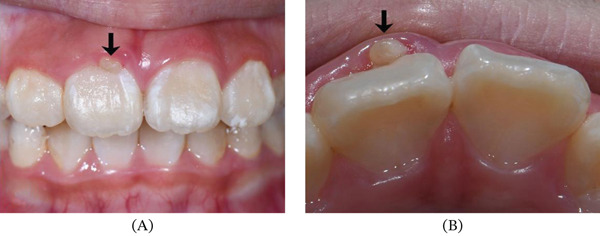
Close‐up clinical photographs of the extra cusp. (A) Anterior view of the triangle‐shape accessory cusp with slight redness of the corresponding gingiva margin. (B) Occlusal view showing fissure between the cusp and the labial surface and the shape of the cusp similar to eagle′ talon.

**Figure 4:**
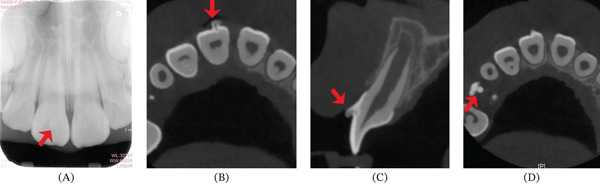
Serial radiographs. (A) Intraoral periapical radiograph of a faintly visible o‐shape opaque image (red arrow) superimposed with the middle crown of the Tooth #11. (B, C) CBCT images with the extra cusp‐like structure probably containing enamel and dentin component according to image density. (B) Axial plane: a small hollow tubercle projected from the labial surface of the Tooth #11 (red arrow); (C) sagittal plane: a small projection with fissure parallel to the labial surface at the junction of crown cervical 1/3 and middle 1/3(red arrow). (D) Red arrow refers to an abnormal central cusp on occlusal surface of the Tooth #15.

Intraoral examination showed mixed dentition and the right incisor had erupted with an accessory small cusp or a slightly raised triangle on the facial aspect extending the length along the axial of the crown, but not reaching the incisal edge (Figure 3a). The cusp was separated from the rest of the crown by a deep groove from occlusal view (Figure 3b). There was an abnormal central cusp on the occlusal surface of the permanent right mandibular second premolar (Figure 2c). Intraoral periapical radiograph showed a small “O”‐shaped radio‐opaque structure superimposing over the normal image of the maxillary right central incisor in the middle of the crown, which was too small to be noticed easily (Figure 4a), and the root had developed to its 3/4 final length with an open trumpet‐shaped foramen. Cone‐beam computed tomography images were obtained for more detailed information. CBCT was performed by the Planmeca ProMax 3D Plus unit (Planmeca Oy, Helsinki, Finland) with these parameters: 80 kVp, 5 mA, voxel size 0.2 mm, field of view of 80∗80 mm. CBCT findings demonstrated a small prominent structure on the labial cervical 1/3 of the maxillary right central incisor, extending approximately 2–3 mm and reaching neither the cementoenamel junction nor incisal edge, with existence fissure parallel to the tooth surface (Figure 4b,c). The cusp consisted of enamel and dentin, which was distinct from the pulp chamber. Interestingly, the germ of the maxillary right first premolar with an abnormal central cusp was seen in CBCT images (Figure 4d). A diagnosis of stage I talon cusp of the maxillary right central incisor was made.

**Figure 5:**
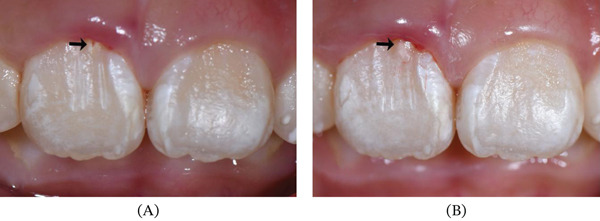
Close‐up clinical photographs of management. (A) A Fissured air bubble encountered in the cervical labial surface of the Tooth #11 after complete cusp removement. (B) Restored by flowable resin.

As stimulation to the gingiva and aesthetic appearance, the talon cusp of the maxillary right central incisor was planed to completely eliminate because of no pulp tissue involved by minimal restorative treatment. After obtaining the informed consent from the child′s parent, the talon cusp was totally reduced using a water‐cooled spray and tapered diamond bur on a high‐speed handpiece, and a fissured air bubble embedded in the labial surface of the Tooth #11 was seen (Figure 5a). Furthermore, flowable resin (3 M Filtek Z350XT, 3 M ESPE, St Paul, Minnesota, United States) was used to restore the minihole and improve esthetic appearance (Figure 5b). Finally, finishing and polishing procedures were performed.

**Figure 6:**
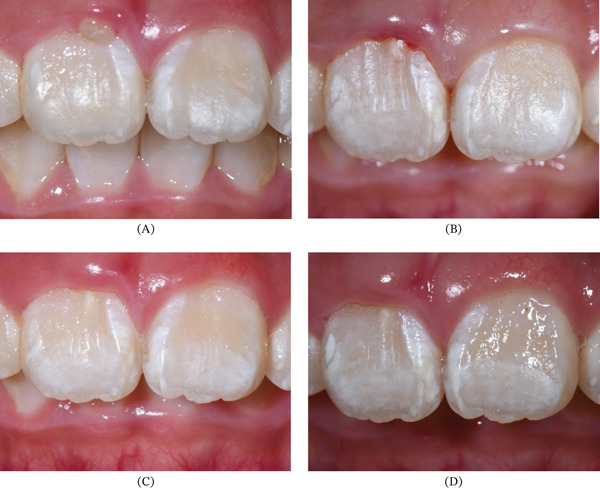
Close‐up clinical photographs of follow‐ups: the corresponding gingiva getting more and more healthy. (A) Pretreatment. (B) Immediately posttreatment. (C) 1‐month follow‐up. (D) 2‐month follow‐up.

**Figure 7:**
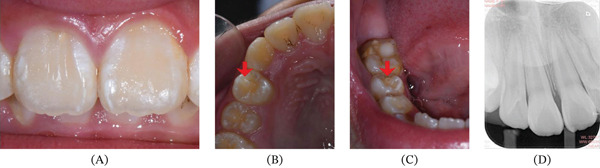
Clinical photographs and radiograph of 18‐month follow‐up. (A) 18‐month follow‐up. (B) #14 after multiple periodic grinding of abnormal central cusp (red arrow). (C) #45 after multiple periodic grinding of abnormal central cusp (red arrow). (D) 18‐month follow‐up periapical X‐ray showing well‐developed root.

The patient was followed up monthly for the first 2 months and the tooth was found to be completely asymptomatic and the gingiva tissue was more and more healthy without complaint of bleeding when toothbrushing (Figure 6a–d). One and a half years′ follow‐up photographs and radiograph showed stability of satisfied appearance and continued root formation of the maxillary right incisor (Figure 7).

## 3. Literature Review and Discussion

**Figure 8:**
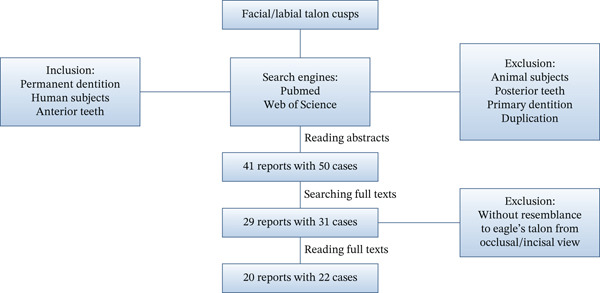
Study design flow chart for the minireview.

The labial talon cusp is extremely rare and only occasionally seen in case reports or series. There is no systematic review on labial talon cusps, probably due to the lack of big data. Although a few systematic reviews or meta analyses targeted on talon cusps [4, 10–14], as we aforementioned, labial and lingual talon cusps might not be simply considered a singular entity; therefore, those reviews which lumped together both may provide little help to comprehend the labial talon cusp. To increase understanding of facial talon cusps in permanent dentition, we searched case reports published in the PubMed/Web of Science databases from January 1990 to July 2025 electronically by using “labial/facial talon cusp” as a search term (Figure 8). A total of 41 reports with 50 cases was selected after reading abstracts to exclude irrelevant reports to labial talon cusp or those that happened in deciduous teeth. Moreover, only 29 reports with 31 cases accessible to get full texts were inclusive. Then all inclusive articles were read to exclude those without resemblance to eagle’s talon from an occlusal/incisal view. Finally, including the present report, there was a total of 20 reports with 22 cases involving 26 teeth seen in Table 2.

**Table 2:** Reported cases of permanent teeth with facial talon cusps seen in literature review.

Serial number	Authors	Year	Nation/place	Gender	Age	Affected tooth (FDI)	Reported type	Associated anomalies/syndromes	Treatment done
1	Mcnamara et al. [15]	1997	Caucasia	Male	13	31‐facial talon	Clinical	—	Extraction and orthodontic treatment
2	de Sousa et al. [16]	1999	—	Female	22	11‐facial talon	Clinical	21‐Palatal talon and 12‐dens invaginatus	Root canal and coronal restoration crown/root longitudinal fracture 4 months later and extraction
3	McKaig et al. [17]	2001	Brimingham	Female	7	11‐facial and palatal talon	Clinical	—	—
4	Oredugba [18]	2005	Nigeria	Male	29	31‐facial talon	Clinical	—	Minimal reduction of talon cusp and APF gel application
5	Llena‐Puy et al. [19]	2005	Valencia	Male	13	32‐facial talon	Clinical	—	—
6	Cubukcu et al. [20]	2006	Turkey	Female	13	11‐germination with labial and palatal talon cusps	Clinical	Abnormal root shape	Extraction of 11 followed by orthodontic correction and prosthetic rehabilitation
7	Mayes [8]	2007	America	—		11‐facial talon	Archeological	—	—
8		2007	America	—		21‐facial talon	Archeological	—	—
9	Sarraf‐Shirazi et al. [21]	2010	Iran	Male	9	11‐one facial cusp and two palatal cusps	Clinical	—	Restorative and preventive therapies
10	Chinni et al. [22]	2012	India	Male	25	11 and 21‐facial talon	Clinical	—	—
11	Hegde et al. [23]	2012	India	Female	25	11‐facial talon	Clinical	—	A yearly periodic recall
12	Nuvvula et al. [24]	2014	India	Male	7	42‐facial talon	Clinical	—	Periodic grinding of the cusp and resin restoration
13	Yazicioglu et al. [2]	2014	Turkey	Female	21	11‐facial talon	Clinical	—	Cuspal grinding and resin restoration
14	Smail‐Faugeron et al. [25]	2016	France	Female	8	12‐gemination with facial and palatal talon	Clinical	—	Extraction and orthodontic treatment
15	Bulut et al. [26]	2017	Caucasia	Male	13	11‐gemination with facial and palatal talon	Clinical	—	Orthodontic, endodontic, and prosthodontic treatment
16	Sudhakar et al. [27]	2017	India	Female	8	11‐three facial cusps 21‐facial cusp	Clinical	—	Refused to undergo treatment
17		2017	India	Male	—	11‐two facial cusps 21‐facial cusp	Clinical	—	Refused to undergo treatment
18	Nandini et al. [28]	2021	India	Female	23	13 and 23‐gemination with facial talon and palatal talon	Clinical	—	Remove of discoloration and GIC restoration
19	Bommanavar et al. [29]	2022	India	Female	10	21‐two facial talon cusps	Clinical	—	Reluctant for treatment
20	Nagpal et al. [30]	2022	India	Female	11	21‐facial and palatal talon	Clinical	22, with facial talon and palatal dens invaginatus 23\31\32, with facial talon	Composite build‐up by grounding the tooth surfaces and resin restoration
21	Arulraj et al. [31]	2023	India	Female	21	11‐two facial talon cusps	Clinical	—	Unwilling for the treatment
22	(The present report)	2025	China	Female	7	11‐trace facial talon	Clinical	14 and 45, with abnormal central cusp	Complete removal once and flowable resin restoration

In summary of the limited data, facial talon cusps were more common in females than males (12 cases to 8, respectively), which was inconsistent with most studies on talon cusps [11–14], but in agreement with a study that summarized previous reports of only labial talon cusps in literature [9]. Furthermore, labial talon cusps in the maxillary permanent dentition showed a statistically significant female bias, whereas in the mandibular show a male bias *(*Fisher′s exact test, *p* = 0.014). As far as dentition type was concerned, labial talon cusps were more common in maxillary than madibular dentition (16 cases to 4, respectively), which agreed with previous studies on talon cusps. With regard to tooth position, the most affected is central incisors (80.8%) followed by lateral incisors (11.5%) and canines (7.7%). This contrasted with the result of those researches mixing up labial and lingual talon cusps together, in which lateral incisors was the most affected tooth follow by centaral incisors [10–14].

Different patterns of occurrence between labial talon cusps in the present and previous studies and talon cusps in reviews may indicate labial and lingual talon cusps are different traits. Five cases (26.3%) were associated with other dental development anomalies and nine cases (47.4%) happened in the India population with the highest rate among various ethnicities, which might suggest a developmental genetic component in etiological factors.

There is scarce research about the occurrence rate of labial talon cusp. To our best knowledge, only one systematic review referred to two cases of labial talon cusp out of 14,400 subjects in turkish population, one in maxillary mesioden and the other one accompanied with palatal talon cusp [11]. Although one study is limited, it can be told, in some extent, how rare the labial talon cusp is from the rate of 2/14,400.Talon cusp can be an independent portent as well as associated with other dental anomalies (e.g., mesiodens, supernumerary teeth, fused teeth, and geminated teeth) and genetic syndromes (e.g. occulo‐facio‐cardio‐dental syndrome) [32–34], so a developmental genetic component in etiology may be indicated. Herein, the present case reported a facial talon cusp of the maxillary right central incisor coexistent with abnormal central cusp of another two tooth on the right side. Abnormal central cusp comprises dentine covered by enamel with or without pulp tissue involved, similar to labial talon cusp but protrusion with wide base from occlusal surface of posterior teeth. This phenomenon may give instinct to further study on etiological mechanism of facial talon cusp.

**Figure 9:**
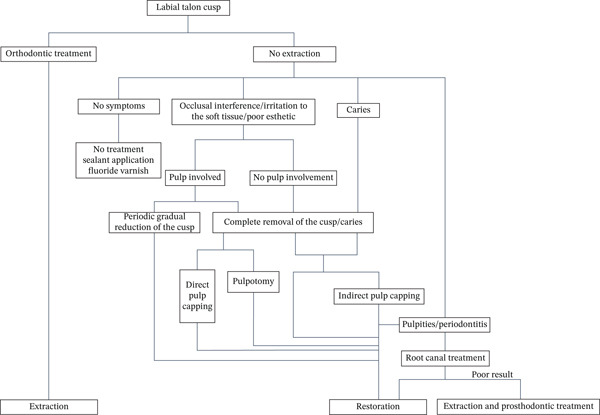
Diagram of therapeutic options for facial talon cusp.

Since the etiology of labial talon cusp remains unclear, usually supposed to be multifactorial, in which genetic factor plays an important role [8, 11], the management of facial talon cusp is to eliminate symptoms or rehabilitate esthetic [15, 16, 35]. If it is asymptomatic, no treatment is needed with only clinical observation, or sealant application and fluoride varnish application can be used to prevent predilection of caries due to fissures. When caries, occlusal interference, esthetic problem, or irritation to lip is caused, the removal of the cusp is considered, which includes sequential grinding to allow sufficient time for formation of reparative dentine and avoid pulp exposure, and complete grinding once followed by assessment of no risk of pulp exposure or irritation by CBCT, or completely grinding once with direct pulp capping or pulpotomy if pulp exposed. Subsequently, composite resin restoration or veneer is placed to recover esthetic appearance. Root canal treatment and following restoration are required once endodontic and periapical diseases progress. Sometimes, patient is going to orthodontic treatment with poor condition of the affected teeth; extraction is also an option. In summary, we proposed a therapeutic diagram for facial talon cusps (Figure 9). Herein, the case presented a trace facial talon cusp with involvement of enamel, few dentin and no pulp tissue told from CBCT, so minimal invasive restoration with flowable resin followed by complete elimination of the cusp once were made and satisfying configuration was achieved.

## 4. Conclusions

The present case reported an extremely rare labial talon cusp on permanent maxillary right central incisor and demonstrated treatment process with good outcome. Thorough knowledge of facial talon cusp is of essence with regards to managing such cases clinically where minimally invasive and preventive principles take first place throughout the whole treatment. Furthermore, herein a minireview on labial talon cusp was made by summarizing previously reported cases.

## Author Contributions

Chuhong Ouyang was the clinician in charge and wrote the main manuscript. Jingwen Chen‐offered good quality and translations of CBCT images and helped in diagnosis. Mingshu Huang‐critically reviewed the article.

## Funding

No funding was received for this manuscript.

## Consent

Consent has been obtained from the patient for publication.

## Conflicts of Interest

The authors declare no conflicts of interest.

## Data Availability

Data sharing is not applicable to this article as no datasets were generated or analyzed during the current study.
